# G protein-coupled estrogen receptor (GPER)/GPR30 forms a complex with the β_1_-adrenergic receptor, a membrane-associated guanylate kinase (MAGUK) scaffold protein, and protein kinase A anchoring protein (AKAP) 5 in MCF7 breast cancer cells

**DOI:** 10.1016/j.abb.2024.109882

**Published:** 2024-01-10

**Authors:** Julia Tutzauer, D. Stephen Serafin, Tobias Schmidt, Björn Olde, Kathleen M. Caron, L.M. Fredrik Leeb-Lundberg

**Affiliations:** a Department of Experimental Medical Science, Lund University, 22184, Lund, Sweden; b Department of Cell Biology and Physiology, University of North Carolina at Chapel Hill, Chapel Hill, NC, 27599, USA; c Wallenberg Center for Molecular Medicine, Department of Clinical Sciences Lund, Division of Pediatrics, Lund University, 22184, Lund, Sweden; d Department of Clinical Sciences, Division of Cardiology, Lund University, 22184, Lund, Sweden

**Keywords:** GPR30, GPER, β_1_-adrenergic receptor, PDZ domain, Breast cancer, Protein complex

## Abstract

G protein-coupled receptor 30 (GPR30), also named G protein-coupled estrogen receptor (GPER), and the β_1_-adrenergic receptor (β1AR) are G protein-coupled receptors (GPCR) that are implicated in breast cancer progression. Both receptors contain PSD-95/Discs-large/ZO-1 homology (PDZ) motifs in their C-terminal tails through which they interact in the plasma membrane with membrane-associated guanylate kinase (MAGUK) scaffold proteins, and in turn protein kinase A anchoring protein (AKAP) 5. GPR30 constitutively and PDZ-dependently inhibits β1AR-mediated cAMP production. We hypothesized that this inhibition is a consequence of a plasma membrane complex of these receptors. Using co-immunoprecipitation, confocal immunofluorescence microscopy, and bioluminescence resonance energy transfer (BRET), we show that GPR30 and β1AR reside in close proximity in a plasma membrane complex when transiently expressed in HEK293. Deleting the GPR30 C-terminal PDZ motif (-SSAV) does not interfere with the receptor complex, indicating that the complex is not PDZ-dependent. MCF7 breast cancer cells express GPR30, β1AR, MAGUKs, and AKAP5 in the plasma membrane, and co-immunoprecipitation revealed that these proteins exist in close proximity also under native conditions. Furthermore, expression of GPR30 in MCF7 cells constitutively and PDZ-dependently inhibits β1AR-mediated cAMP production. AKAP5 also inhibits β1AR-mediated cAMP production, which is not additive with GPR30-promoted inhibition. These results argue that GPR30 and β1AR form a PDZ-independent complex in MCF7 cells through which GPR30 constitutively and PDZ-dependently inhibits β1AR signaling via receptor interaction with MAGUKs and AKAP5.

## 1. Introduction

GPR30, or G protein-coupled estrogen receptor (GPER), is a G protein-coupled receptor (GPCR) that is attracting attention as a putative prognostic marker and drug target in breast cancer. The receptor was originally reported to bind 17β-estradiol (E2) and mediate rapid nongenomic estrogenic responses [1,2], and the synthetic compound G-1 was subsequently described as a specific GPR30 agonist [3]. However, numerous subsequent studies have failed to validate these results [4–12], questioning the use of these agents to target GPR30. On the other hand, GPR30 exhibits ligand-independent constitutive activity [8, 10,11] and constitutively regulates other GPCR, including the β_1_-adrenergic receptor (β1AR) [8], follicle-stimulating hormone receptor (FSHR) [13], and kisspeptin receptor (Kiss1R) [14].

GPR30 has been studied in relation to breast cancer prognosis, where specifically plasma membrane-localized GPR30 predicts worse disease outcome [15,16]. The subcellular distribution of GPR30 is complex, localizing both in intracellular membranes and the plasma membrane, the latter which is typical of GPCR. Plasma membrane localization of GPR30 and constitutive receptor activity are both favored by an interaction between the receptor C-terminal PSD-95/Discs-large/ZO-1 homology (PDZ) motif and a membrane-associated guanylate kinase (MAGUK) scaffold proteins and the MAGUK-associated protein kinase A anchoring protein (AKAP) 5 [8,13]. Two MAGUK proteins have been shown to interact with the GPR30, including postsynaptic density-95 (PSD-95) [8,17,18] and synapse-associated protein 97 (SAP97) [8,19]. While GPR30 is expressed and has been studied in breast cancer cells, primarily in ER-positive MCF7 cells and ER-negative SkBr3 cells [20], the above mechanism has not yet been addressed in such systems.

The sympathetic nervous system innervates the human mammary gland [21]. While the role of this system in breast cancer is complex, preclinical studies in animal models suggest that sympathetic stimulation promotes tumor progression primarily through a β_2_-adrenergic receptor (β2AR) subtype [22]. Few, if any studies have been done regarding the role or prognostic value specifically of the β1AR subtype in breast cancer. However, it is interesting to note that β1AR-selective antagonists used to treat hypertension dose-dependently increase the risk of developing breast cancer in hypertensive patients [23].

β1AR is the predominant βAR subtype expressed in MCF7 and SkBr3 breast cancer cells (data available from v19.3; www.proteinatlas.org [24]). Like GPR30, β1AR also interacts via a C-terminal PDZ motif with MAGUK scaffold proteins, including PSD-95 [25,26] and Sap97 [25–27], and in turn with AKAP5 [27,28], the latter which regulates plasma membrane anchoring and agonist-stimulated signaling also of this receptor [27–29]. GPR30 constitutively inhibits β1AR-mediated cAMP production through a pertussis toxin-insensitive mechanism that is dependent on the GPR30 C-terminal PDZ motif [8]. We hypothesized that GPR30 and β1AR form a plasma membrane complex in breast cancer cells through which GPR30 negatively regulates β1AR signaling. Indeed, this could be a mechanism by which GPR30 constitutively regulates adrenergic signaling in breast cancer and possibly other pathophysiological systems.

## 2. Material and methods

### 2.1. Cell culture and DNA constructs

HEK293, MCF7, SkBr3, T47D, and MDA-MB-231 cells (American Type Culture Collection, Manassas, VA) were grown in Dulbecco’s Modified Eagle’s Medium (DMEM) without pyruvate, DMEM supplemented with pyruvate and L-glutamine, McCoy’s Modified 5a medium, RPMI-1640 medium, and DMEM supplemented with pyruvate and L-glutamine, respectively, all supplemented with 10 % fetal bovine serum (FBS) and 5 % penicillin/streptomycin. All cell lines were cultivated in 5 % CO_2_ at 37 °C in a humidified incubator.

N-terminally FLAG- and HA-tagged human GPR30 in pcDNA3.1 and a GPR30 construct, in which the four C-terminal residues in GPR30 (-Ser-Ser-Ala-Val) were deleted (GPR30ΔSSAV), in pcDNA3.1 were made as previously described [8]. CD33-CMV-Myc-GPR30-rLuc, CMV-RAMP3-eYFP, and CMV-eYFP plasmid constructs were made as previously described [30]. FLAG-tagged human β1AR in pcDNA3 was a gift from Dr. Robert Lefkowitz (Addgene plasmid #14698; http://n2t.net/addgene:14698; RRID: Addgene_14698) [31]. β1AR-eYFP and GPR30ΔSSAV-rLuc in pcDNA3.1 were synthesized by Biomatik USA (Wilmington, DE). The KRAS-Venus plasmid construct was kindly donated by Dr. Nevin A. Lambert (Medical College of Georgia, Augusta University, GA), the CXCR4-eYFP plasmid construct by Dr. Nikolaus Heveker (CHU Sainte-Justine Research Center at Montreal, Quebec, Canada), and the pEGFP-N1 AKAP79 plasmid construct by Dr. John D. Scott (University of Washington School of Medicine, WA).

TransIT-LT1 (Mirus Bio LLC, Madison, WI) was used to transiently transfect plasmid DNA in HEK293 cells (3 μl/μg DNA for 48 h) and MCF7 cells (2 μl/μg DNA for 72 h), except with HEK293 cells used in the BRET^1^ assay, which were transfected using Lipofectamine 2000 (Invitrogen, Waltham, MA). Cells transiently transfected with plasmid containing receptor construct were always compared to cells transfected with empty plasmid alone (Mock).

### 2.2. Immunoprecipitation and immunoblotting

Immunoprecipitation and immunoblotting were done as previously described [8]. Proteins were immunoprecipitated with goat GPR30 antibody (Ab) (R&D Systems Minneapolis, MN), rabbit β1AR Ab (Santa Cruz Biotechnology, Dallas, TX), pan-MAGUK Ab (Merck Millipore, Billerica, MA), or mouse AKAP5 Ab (BD Biosciences, San Jose, CA) coupled to protein G-Sepharose (GE Healthcare, Little Chalfont). Proteins were immunoblotted with goat GPR30 Ab (1:200), rabbit β1AR Ab (1:1000), mouse pan-MAGUK Ab (1:1000), mouse AKAP5 Ab (1:1000), or mouse GAPDH Ab (Sigma Aldrich; 1:1000).

### 2.3. Immunofluorescence microscopy

Immunofluorescence microscopy of HEK293 cells was done as previously described [8]. In short, HEK293 cells were fixed and permeabilized and then incubated with mouse M1 FLAG Ab (Sigma Aldrich; 1:500) for 1 h at 22 °C or goat GPR30 Ab (1:100) overnight at 4 °C. Receptors were then visualized by incubating the fixed cells with secondary Alexa488-labeled goat Ab or mouse IgG2b Ab (Life Technologies). Images were collected using a Nikon Eclipse TE2000 confocal fluorescence microscope.

### 2.4. Flow cytometry analysis of cell surface receptors

Cell surface GPR30 and β1AR in MCF7 cells were analyzed by flow cytometry. Cells were detached by trypsination, washed with PBS, and fixed with 2 % paraformaldehyde for 15 min. After washing with PBS, cells were suspended in PBS with 0.5 % BSA. Each sample was split into two, where one was incubated without primary Ab and the other with either goat GPR30 Ab (1:100) or rabbit β1AR Ab (1:100) overnight at 4 °C. The following day, all samples were rinsed with PBS and incubated with APC-labeled anti-goat Ab (R&D Systems Minneapolis, MN; 1:500) or secondary Alexa488-labeled goat anti-rabbit Ab (1:1000) for 20 min at room temperature. After a final wash in PBS, the cells were resuspended in PBS, and samples were acquired using a CytoFLEX flow cytometer (Beckman Coulter) and analyzed using the CytExpert software (v2.3, Beckman Coulter). Forward and side scatter measurements were attained with gain settings in linear mode.

### 2.5. cAMP production

Production of cAMP in MCF7 cells was monitored using the GloSensor cAMP system according to the manufacturer’s instructions (Promega, Madison, WI). Briefly, 80 % confluent cells grown in 6-well plates were transfected with 2 μg GloSensor plasmid and 1 μg of each additional plasmid per well and then incubated for 48 h before being seeded in 96-well plates (25,000 cells/well) over night. The cells were then incubated with the GloSensor cAMP reagent in phenol red-free DMEM for 2 h in the dark at room temperature. Prior to assay, the cells were treated with 25 μM rolipram in the dark at room temperature for 10 min. Luminescence was read before and after addition of stimulus using a Clariostar luminometer.

### 2.6. Bioluminescence resonance energy transfer (BRET) assay

A BRET^1^ assay was used to monitor the interaction between GPR30 and β1AR. The following constructs were used: Myc-GPR30-rLuc, Myc-GPR30ΔSSAV-rLuc, RAMP3-eYFP, β1AR-eYFP, eYFP, KRAS-Venus, and CXCR4-eYFP. As a positive control, we assessed the interaction of GPR30-rLuc with RAMP3-eYFP [30], and as negative controls, we assessed the interactions between GPR30-rLuc and eYFP, GPR30-rLuc and KRAS-Venus, and GPR30-rLuc and CXCR4-eYFP. A constant concentration of GPR30-rLuc (0.5 μg/well) was used in conjunction with serially-increased concentrations of acceptor-YFP/Venus (max concentration of 2.5 μg/well).

For the BRET^1^ assay, HEK293T cells were seeded (35,000 cells/well) into a white-walled 96-well plate (Corning, Corning, NY) in DMEM (Corning, Corning, NY) supplemented with 10 % FBS (Avantor Seradigm) and 1 % penicillin-streptomycin (Gibco) and grown in 5 % CO_2_ at 37 °C. The following day, media was replaced with Opti-MEM (ThermoFisher Scientific) and incubated for 2 h in 5 % CO_2_ at 37 °C. Then, GPR30-rLuc and acceptor-YFP/Venus DNA were transfected into cells using Lipofectamine 2000. The following day, media was replaced with 1x PBS (Gibco) supplemented with calcium and magnesium as well as 5 μM coelenterazine-h (benzyl-coelenterazine) (Promega, Madison, WI). Ten minutes later, the total YFP/Venus fluorescence (for cells that did not receive coelenterazine-h), coelenterazine-h emission (~480 nM) and eYFP/Venus emission (~530 nM) were read on a Cytation 5 multi-mode plate reader (Agilent, Santa Clara, CA). Data was graphed as acceptor (-eYFP/Venus)/donor (GPR30-rLuc/GPR30ΔSSAV-rLuc) ratio (Y-axis) versus total eYFP/Venus fluorescence/luminescence (X-axis). Curves were fitted and analyzed using both a non-linear regression with one-site specific binding and linear regression using GraphPad Prism program (GraphPad, La Jolla, CA). These values were utilized to determine interaction strength, based on previously published criteria [32]; Bmax < 0.1 = No interaction, Bmax > 0.1 and Linear R^2^ > hyperbolic R^2^ = Poor interaction, and B_max_ > 0.1 and Linear R^2^ < hyperbolic R^2^ = Good interaction.

### 2.7. Data analysis

Data are presented as means with error bars representing SD, SEM, or 95 % confidence interval. For statistical comparison between two groups, Student’s t-test was applied for parametrical data, and Mann-Whitney *U* test for non-parametrical data. For comparisons between multiple groups, one-way ANOVA with Bonferonni’s post hoc was used. *P*-values less than 0.05 were considered statistically significant. Data analysis was performed using the GraphPad Prism program.

## 3. Results

### 3.1. GPR30 and β1AR form a PDZ-independent complex in HEK293 cells

**Fig. 1.**
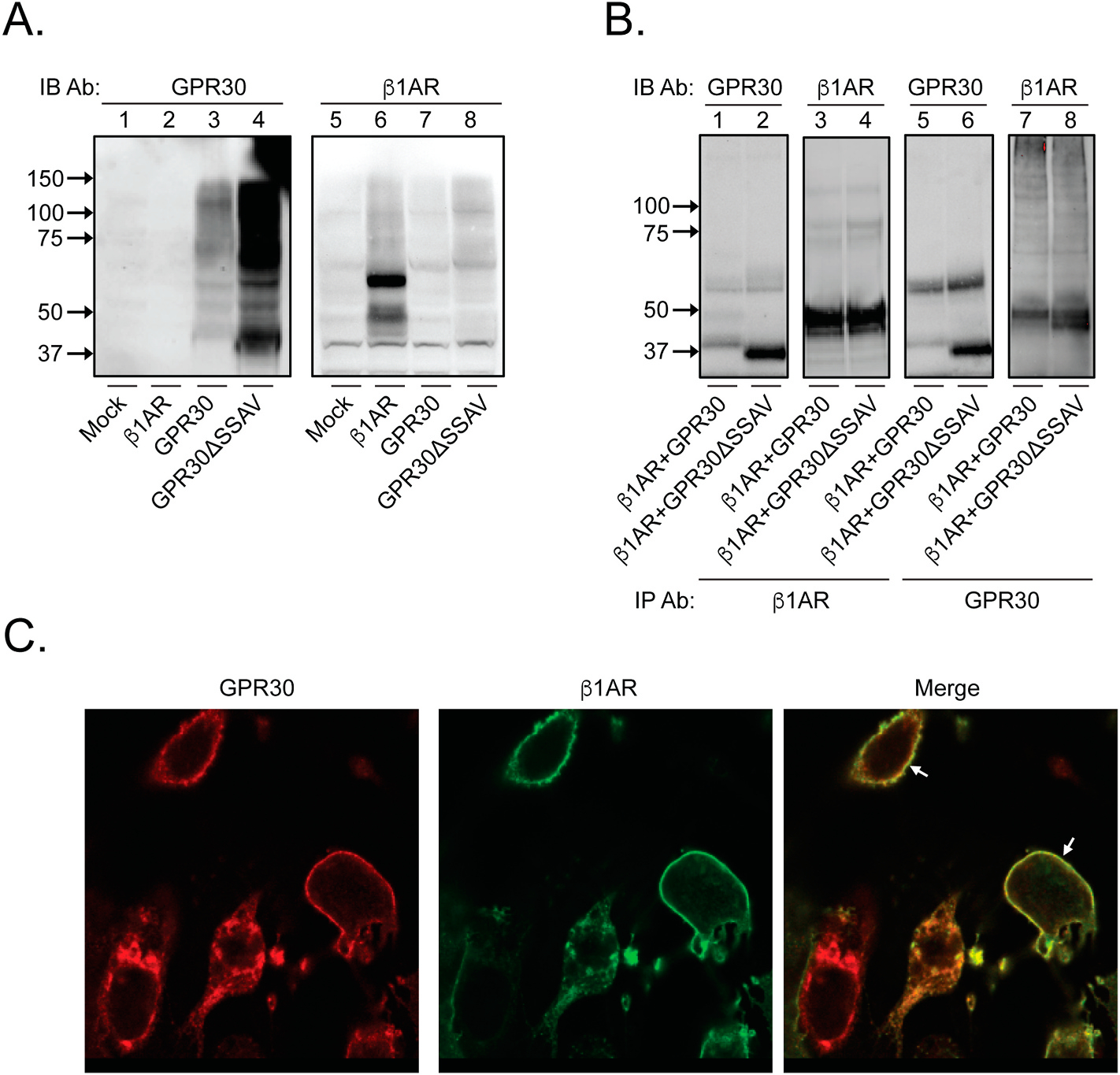
GPR30 and β1AR form a PDZ-independent complex in HEK293. In panel **A**, lysates of HEK293 cells transiently transfected with empty pcDNA3 plasmid (*Mock*) or plasmids containing β1AR, GPR30, or GPR30ΔSSAV were immunoblotted with GPR30 Ab (lanes 1–4) or β1AR Ab (lanes 5–8). In panel **B**, lysates of HEK293 cells transiently transfected with a combination of plasmids containing β1AR and GPR30 (lanes 1, 3, 5, and 7) or β1AR and GPR30ΔSSAV (lanes 2, 4, 6, and 8) were immunoprecipitated with β1AR Ab (lanes 1–4) or GPR30 Ab (lanes 5–8) and then immunoblotted with GPR30 Ab (lanes 1, 2, 5, and 6) or β1AR Ab (lanes 3, 4, 7, and 8). In panel **C**, HEK293 cells transiently transfected with GPR30 and β1AR were fixed and stained with GPR30 and M1 FLAG Abs. The images were collected using a Nikon Eclipse confocal microscope, 60× objective, 50 μm zoom. The results are representative of experiments performed at least three times. In panels **A** and **B**, molecular mass standards (*left side*) are indicated, and in panel **C**, receptor colocalization is indicated (*arrows*).

Investigating if native GPR30 and β1AR exist in a complex in cells requires the use of highly specific receptor Abs. To confirm the specificity of the receptor Abs used in this study, we first immunoblotted receptors transiently expressed individually in HEK293 cells, a well-defined GPCR cell model system lacking significant native GPR30 and β1AR expression. GPR30 was detected with a polyclonal goat GPR30 Ab raised against the extracellular N-terminal domain of the receptor, which we previously reported to be highly specific for the receptor [11, 33]. Fig. 1A shows that this Ab recognized GPR30 (lane 3) and GPR30ΔSSAV (lane 4), a truncated GPR30 construct that lacks the C-terminal PDZ motif, whereas the Ab did not recognize β1AR (lane 2). Specific GPR30 species were observed at about 40 kDa, the theoretical molecular mass of the receptor, and at several higher masses, which are most likely post-translationally modified receptor species and detergent-resistant receptor oligomers. In contrast, a polyclonal rabbit β1AR Ab recognized β1AR (lane 6) but not GPR30 (lane 7) or GPR30ΔSSAV (lane 8), and primarily two β1AR species were observed at about 50 kDa and 60 kDa. Consequently, these Abs were considered specific for each receptor.

**Fig. 2.**
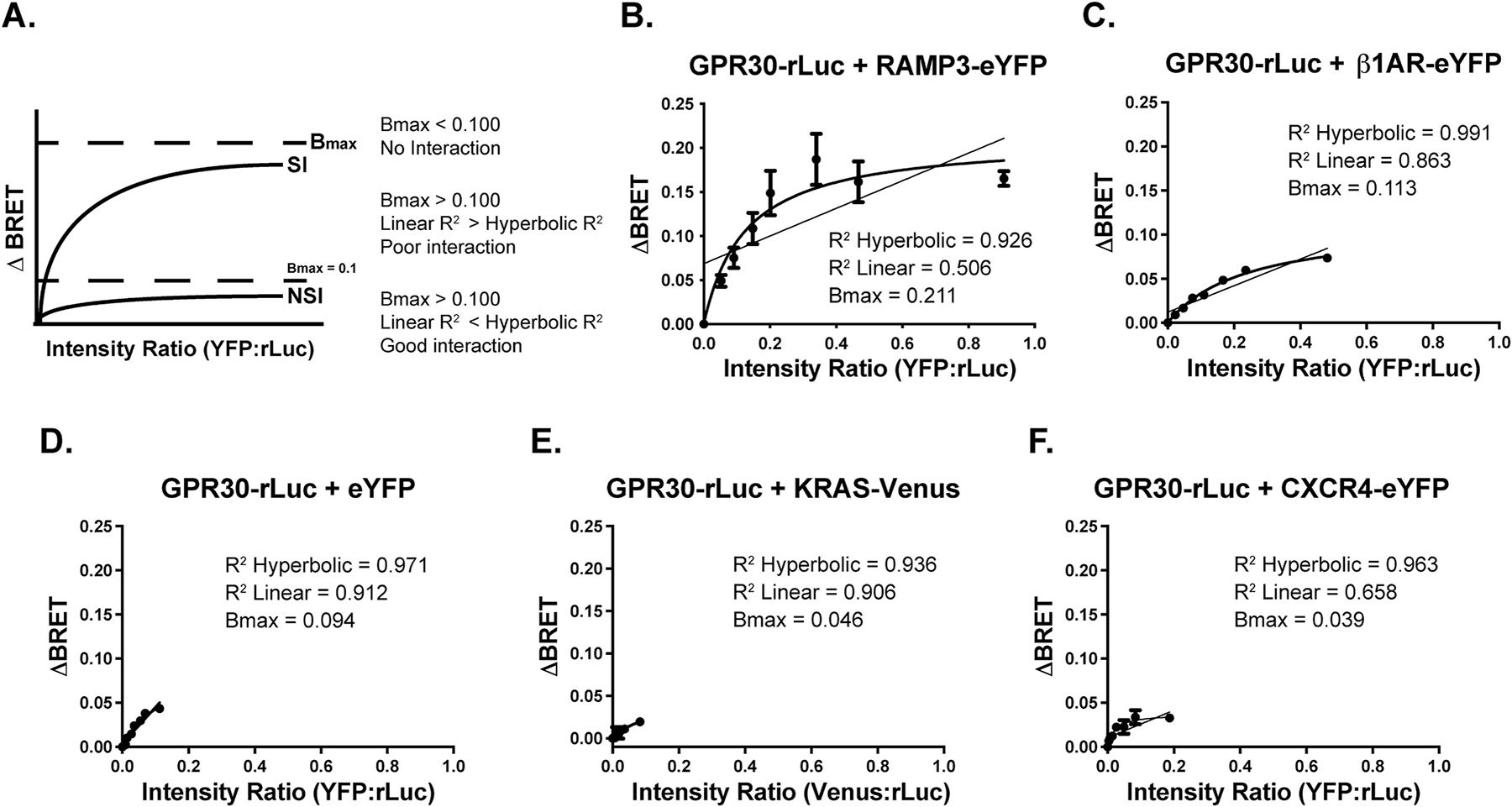
BRET^1^ analysis validates the close proximity of GPR30 and β1AR. ΔBRET was determined using HEK293T cells co-expressing GPR30-rLuc and β1AR-eYFP. GPR30 interaction with RAMP3-eYFP was used as positive control, and GPR30-rLuc interactions with and KRAS-Venus and CXCR4-YFP were used as negative controls. In panel **A**, previously published criteria that were utilized to delineate specific interactions (SI) from non-specific interactions (NSI) are shown: B_max_ < 0.1 = No interaction (NSI), B_max_ > 0.1 and Linear R^2^ > hyperbolic R^2^ = Poor interaction, and B_max_ > 0.1 and Linear R^2^ < hyperbolic R^2^ = Good interaction. In panel **B,** BRET^1^ analysis of GPR30 and RAMP3-YFP is shown, revealing a good interaction (positive control) between GPR30 and RAMP3 (B_max_ = 0.211, R^2^ hyperbolic = 0.926, R^2^ linear = 0.506). In panel **C**, BRET^1^ analysis of GPR30 and β1AR is shown, revealing a good interaction between the GPR30 and β1AR (B_max_ = 0.113, R^2^ hyperbolic = 0.991, R^2^ linear = 0.863). In panel **D,** BRET^1^ analysis of GPR30 and eYFP is shown, revealing no interaction (negative control) (B_max_ = 0.094, R^2^ hyperbolic = 0.971, R^2^ linear = 0.912). In panel **E**, BRET^1^ analysis of GPR30 and KRAS-Venus is shown, revealing no interaction (negative control) (B_max_ = 0.046, R^2^ hyperbolic = 0.936, R^2^ linear = 0.906). In panel **F**, BRET^1^ analysis of GPR30 and CXCR4-YFP is shown, revealing no interaction (negative control) (B_max_ = 0.039, R^2^ hyperbolic = 0.963, R^2^ linear = 0.658). Data is graphed as the acceptor (-eYFP/Venus)/donor (GPR30-rLuc) ratio (Y-axis) versus total eYFP/Venus fluorescence/luminescence (X-axis), and the curve was fitted utilizing a non-linear regression with one-site specific binding. Data is shown as mean ± SEM of three independent experiments.

To begin to address if GPR30 and β1AR exist in a complex, we first performed receptor co-immunoprecipitation from lysates of HEK293 cells co-expressing the receptors. Fig. 1B (lanes 1, 3, 5, and 7) shows that immunoprecipitation with the β1AR Ab (lanes 1 and 3) or the GPR30 Ab (lanes 5 and 7) yielded co-precipitation of the two receptors, with β1AR observed primarily as a 50-kDa species and GPR30 primarily as 40-kDa and 55-kDa species. These results suggest that the two receptors are in close proximity when expressed in HEK293 cells. To address the subcellular localization of a putative receptor complex, we used confocal immunofluorescence microscopy to view fixed HEK293 cells co-expressing HA-tagged GPR30 and F-β1AR and stained with the GPR30 Ab and M1 FLAG Ab, respectively. As shown in Fig. 1C, the two receptors at least in part co-localize at the plasma membrane. Finally, we addressed a receptor complex using BRET with HEK293 cells co-expressing GPR30-rLuc and β1AR-eYFP. Co-expression of GPR30-rLuc with RAMP3-eYFP was used as a positive control [30], and eYFP, KRAS-Venus, a distinct plasma membrane-associated protein, and CXCR4-eYFP, a distinct plasma membrane GPCR, were used as negative controls. Fig. 2A shows previously published criteria that were utilized to delineate specific interactions (SI) from non-specific interactions (NSI): Bmax < 0.1 = No interaction (NSI), Bmax > 0.1 and Linear R^2^ > hyperbolic R^2^ = Poor interaction, and B_max_ > 0.1 and Linear R^2^ < hyperbolic R^2^ = Good interaction [32]. As shown in Fig. 2B, BRET^1^ analysis of GPR30-rLuc and RAMP3-YFP reveals a good interaction (positive control) between GPR30 and RAMP3 (B_max_ = 0.211, R^2^ hyperbolic = 0.926, R^2^ linear = 0.506). Analysis of GPR30-rLuc and β1AR-YFP also reveals a good interaction between the GPR30 and β1AR (B_max_ = 0.113, R^2^ hyperbolic = 0.991, R^2^ linear = 0.863) (Fig. 2C). On the other hand, analysis of GPR30-rLuc and eYFP (B_max_ = 0.094, R^2^ hyperbolic = 0.971, R^2^ linear = 0.912) (Fig. 2D), KRAS-Venus (B_max_ = 0.046, R^2^ hyperbolic = 0.936, R^2^ linear = 0.906) (Fig. 2E) and CXCR4-eYFP (B_max_ = 0.039, R^2^ hyperbolic = 0.963, R^2^ linear = 0.658) (Fig. 2F) reveal no interaction (negative control). Thus, GPR30 and β1AR are in close proximity (<10 nm) when expressed in HEK293 cells. Together, these results argue that GPR30 and β1AR form a complex in these cells.

Previous studies show that GPR30 and β1AR each interact PDZ-dependently with MAGUK scaffold proteins at the plasma membrane [8,17–19,25–29]. To address if the putative receptor complex requires PDZ-dependent interactions, we first immunoprecipitated lysates of HEK293 cells co-expressing β1AR and GPR30ΔSSAV, which lacks the C-terminal PDZ motif (Fig, 1B, lanes 2, 4, 6, 8). Immunoprecipitation with the β1AR Ab (Fig. 1B, lanes 2 and 4) or the GPR30 Ab (lanes 6 and 8) yielded co-precipitation of the two receptors. We also evaluated the interaction between GPR30ΔSSAV-rLuc and β1AR-YFP with BRET, using the interaction with RAMP3 as a positive control and with KRAS-Venus as a negative control. Good interactions were observed between GPR30ΔSSAV and RAMP3 (B_max_ = 0.207, R^2^ hyperbolic = 0.953, R^2^ linear = 0.451) and between GPR30ΔSSAV and β1AR (B_max_ = 0.200, R^2^ hyperbolic = 0.987, R^2^ linear = 0.982), whereas no interaction was observed between GPR30ΔSSAV and KRAS (B_max_ = incalculable, R^2^ hyperbolic = 0.719, R^2^ linear = 0.782) (Supplementary Fig. 1). Thus, the receptor complex does not appear to be PDZ-dependent.

### 3.2. MCF7 cells express MAGUKs, AKAP5, GPR30, and β1AR in the plasma membrane

**Fig. 3.**
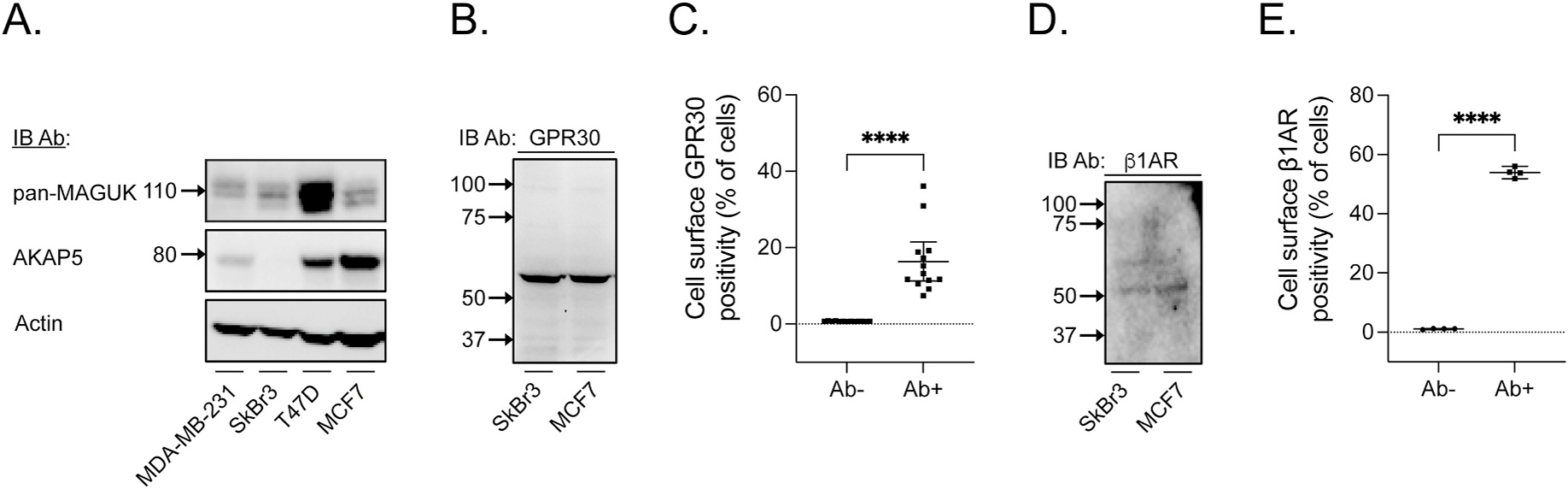
MCF7 cells express GPR30, β1AR, MAGUKs, and AKAP5. In panel **A**, lysates of MCF7, SkBr3, T47D, and MDA-MB-231 cells were immunoblotted with pan-MAGUK Ab, AKAP5 Ab, or β-actin Ab. In panel **B**, lysates of MCF7 and SkBr3 cells were immunoblotted with GPR30 Ab. In panel **C**, flow cytometry was performed on non-permeabilized MCF7 cells with and without GPR30 Ab, and the results presented as percent positive cells. In panel **D**, lysates of MCF7 and SkBr3 cells were immunoblotted with β1AR Ab. In panel **E**, flow cytometry was performed on non-permeabilized MCF7 cells with and without β1AR Ab, and the results are presented as percent positive cells. In panels **C** and **E**, data is shown as median with 95 % CI.

To address if native GPR30 and β1AR reside in a plasma membrane complex with MAGUK and AKAP5 in breast cancer cells, we first searched for a breast cancer cell line that express all four proteins. To this end, four cell lines in which GPR30 has been previously studied, including ER-positive MCF7 and T47D cells, ER-negative SkBr3 cells, and triple-negative MDA-MB-231, were immunoblotted for MAGUK proteins and AKAP5. Fig. 3A shows that all the cell lines express MAGUK proteins, whereas AKAP5 is primarily expressed in ER-positive T47D and MCF7 cells, with little to no expression in ER-negative SkBr3 cells. Consequently, we focused our attention primarily on MCF7 cells. Immunoblotting of MCF7 and SkBr3 cell lysates with the GPR30 Ab shows that these cells express GPR30, which was observed primarily as the 55-kDa species under these conditions, but also with several minor species both above and below this molecular mass (Fig. 3B). To determine the percentage of cells expressing GPR30 in the plasma membrane of MCF7 cells, flow cytometry of non-permeabilized cells with the GPR30 Ab was compared to matched samples without primary Ab. A mean of 16.36 % (lower 95 % CI = 11.24, upper 95 % CI = 21.49) of the cells incubated with primary GPR30 Ab were GPR30 positive, as compared to a mean of 0.71 % (lower 95 % CI = 0.6718, upper 95 % CI = 0.7482) with the primary Ab-negative reference samples (Fig. 3C). MCF7 cells and SkBr3 cells also express β1AR, migrating primarily as the 50-kDa and 60-kDa species (Fig. 3D). Flow cytometry of non-permeabilized MCF7 cells revealed a mean of 53.93 % (lower 95 % CI = 51.21, upper 95 % CI = 56.65) of the cells with the β1AR Ab, as compared to a mean of 1.08 % (lower 95 % CI = 0.7777, upper 95 % CI = 1.377) with the primary Ab-negative reference samples (Fig. 3E). Thus, MCF7 cells express statistically significant amounts of both GPR30 and β1AR in the plasma membrane.

**Fig. 4.**
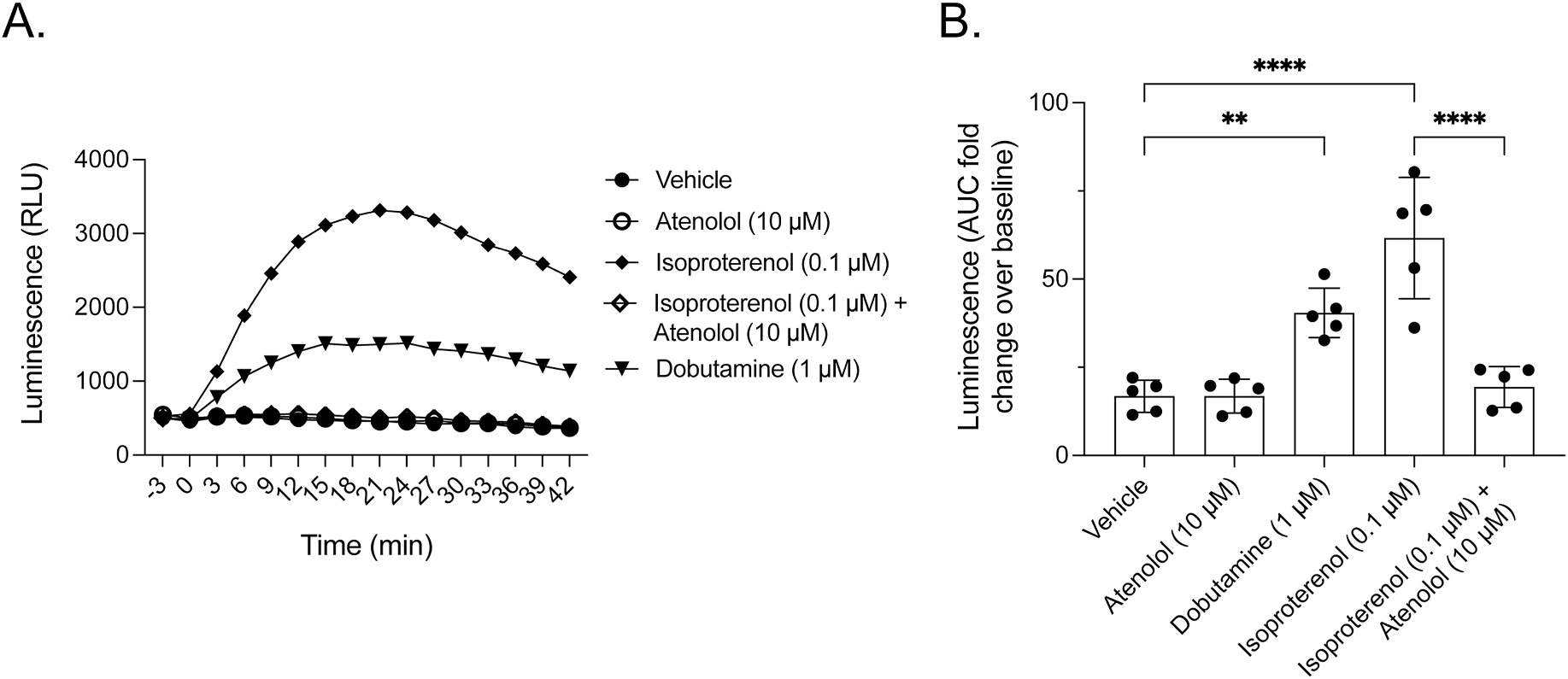
MCF7 cells express functional β1AR. In panel **A**, MCF7 cells were treated with various adrenergic receptor agonists and antagonists as indicated. cAMP was measured in real time for luminescence with the GloSensor assay and presented as RLU. The result is representative of at least 3 experiments. In panel **B**, AUC was calculated for each treatment in panel **A**. The results are representative of at least 3 experiments with each data point being the mean ± SD. **, p < 0.01; ****, p < 0.001.

The Human Protein Atlas database reports that MCF7 cells express β1AR mRNA but no detectable β2AR or β3AR mRNA expression (data available from v19.3; www.proteinatlas.org [24]). To confirm that MCF7 cells express functional β1AR, we monitored G protein-mediated cAMP production. As shown in Fig. 4, the non-specific βAR agonist isoproterenol (0.1 μM) and the specific β1AR partial agonist dobutamine (1 μM) elicited rapid (panel A) and statistically significant rises in cAMP production (panel B), and the isoproterenol response was completely inhibited by the specific β1AR antagonist atenolol (10 μM). Thus, our results confirm those in the Human Protein Atlas database that MCF7 cells express a β1AR subtype. Together, these results show that MCF7 cells express all the proteins of interest, including GPR30, β1AR, MAGUKs, and AKAP5.

### 3.3. Native GPR30 and β1AR form a plasma membrane complex with MAGUKs and AKAP5 in MCF7 cells

**Fig. 5.**
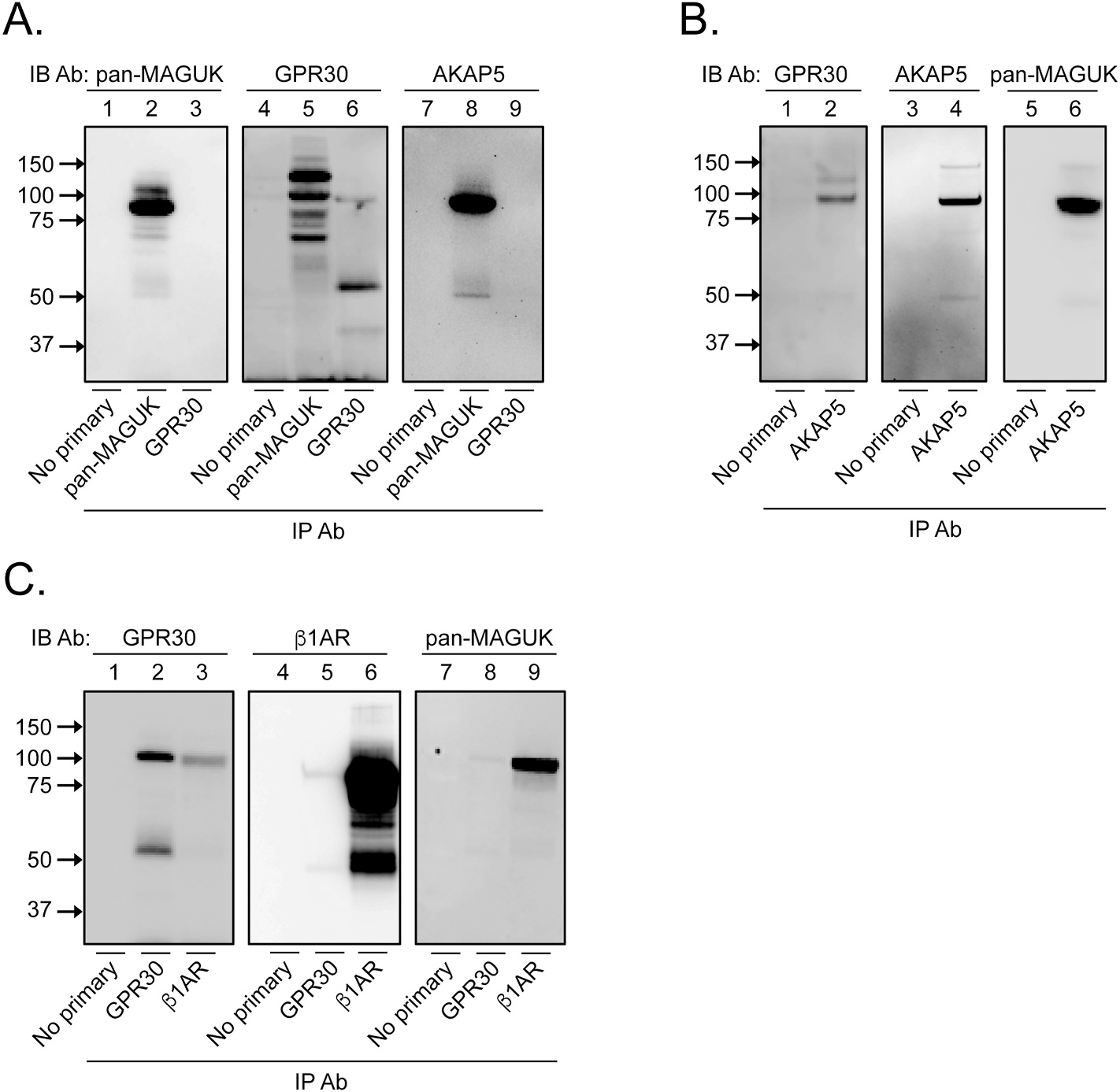
GPR30, β1AR, MAGUKs, and AKAP5 form a complex in MCF7 cells. In panel **A**, MCF7 lysates were immunoprecipitated with or without primary pan-MAGUK Ab or GPR30 Ab and immunoblotted with pan-MAGUK Ab (lanes 1–3), the blot stripped and re-blotted with GPR30 Ab (lanes 4–6), and the blot stripped again and re-blotted with AKAP5 Ab (lane 7–9). In panel **B**, MCF7 lysates were immunoprecipitated with or without primary AKAP5 Ab and immunoblotted with GPR30 Ab (lanes 1–2), the blot stripped and re-blotted with AKAP5 Ab (lanes 3–4), and the blot stripped again and re-blotted with pan-MAGUK Ab (lane 5–6). In panel **C**, MCF7 lysates were immunoprecipitated with or without primary GPR30 or β1AR Ab and immunoblotted with GPR30 Ab (lanes 1–3), the blot stripped and re-blotted with β1AR Ab (lanes 4–6), and the blot stripped again and re-blotted with pan-MAGUK Ab (lane 7–9). The results are representative of at least 3 experiments.

To address if a native complex of GPR30, β1AR, a MAGUK, and AKAP5 exists in MCF7 cells, we performed a series of immunoprecipitation experiments with Abs against each protein. In one set of experiments (Fig. 5A), pan-MAGUK and GPR30 Ab immunoprecipitates were blotted with pan-MAGUK Ab (lanes 1–3), the blot stripped and re-blotted with GPR30 Ab (lanes 4–6), and the blot stripped again and re-blotted with AKAP5 Ab (lane 7–9). As expected, the pan-MAGUK Ab precipitated MAGUK species at 90–110 kDa (Fig. 5A, lane 2), and the GPR30 Ab precipitated the previously identified GPR30 species at 40-kDa, 55-kDa, and about 100-kDa (lane 6) (cf. Fig. 1A, lane 3). Fig. 5A, lane 5 shows that the pan-MAGUK Ab co-precipitated several of the previously observed GPR30 species at 70–120 kDa (cf. Fig. 1A, lane 3) as well as an AKAP5 species at about 100 kDa (lane 8). Curiously, no clear MAGUK or AKAP5 species were observed in the GPR30 Ab immunoprecipitate in this blot (Fig. 5A, lanes 3 and 9). In a second set of stripping and reblotting experiments (Fig. 5B), AKAP5 Ab immunoprecipitates were blotted with GPR30 Ab (lanes 1–2), the blot stripped and re-blotted with AKAP5 Ab (lanes 3–4), and the blot stripped again and re-blotted with MAGUK Ab (lane 5–6). As expected, the AKAP5 Ab precipitated AKAP5 (Fig. 5B, lane 4) and co-precipitated MAGUK species (lane 6). The AKAP5 Ab also co-precipitated GPR30 species at about 100 kDa and 120 kDa (Fig. 5B, lane 2), similar to that observed in the pan-MAGUK immunoprecipitate (cf. Fig. 5A, lane 5). In a third set of experiments (Fig. 5C), GPR30 and β1AR Ab immunoprecipitates were blotted with GPR30 Ab (lanes 1–3), the blot stripped and re-blotted with β1AR Ab (lanes 4–6), and the blot stripped again and re-blotted with pan-MAGUK Ab (lane 7–9). Fig. 5C shows that the β1AR Ab precipitated β1AR (lane 6) and co-precipitated MAGUK species (lane 9) and a GPR30 species at about 100 kDa (lane 3). Furthermore, the GPR30 Ab precipitated the previously observed GPR30 species at about 55 kDa and 100 kDa (Fig. 5C, lane 2) and co-precipitated β1AR (lane 5) and MAGUK species (lane 9). These results argue that GPR30 exists in a complex with β1AR, MAGUKs, and AKAP5 also in native MCF7 cells. As observed, the GPR30 Ab was relatively less efficacious at co-precipitating some of the other components of the proposed complex (see e.g. Fig. 5A, lanes 3 and 9, Fig. 5C, lanes 5 and 8). This lower efficacy may be explained by that the GPR30 Ab either is a less avid immunoprecipitating Ab or that GPR30 is primarily localized intracellularly in cells, the latter resulting in a GPR30 Ab precipitate with a relatively lower amount of plasma membrane-localized GPR30, the form interacting with plasma membrane-localized β1AR, MAGUKs, and AKAP5.

### 3.4. GPR30 inhibits β1AR-mediated cAMP production through a PDZ-dependent mechanism in MCF7 cells

**Fig. 6.**
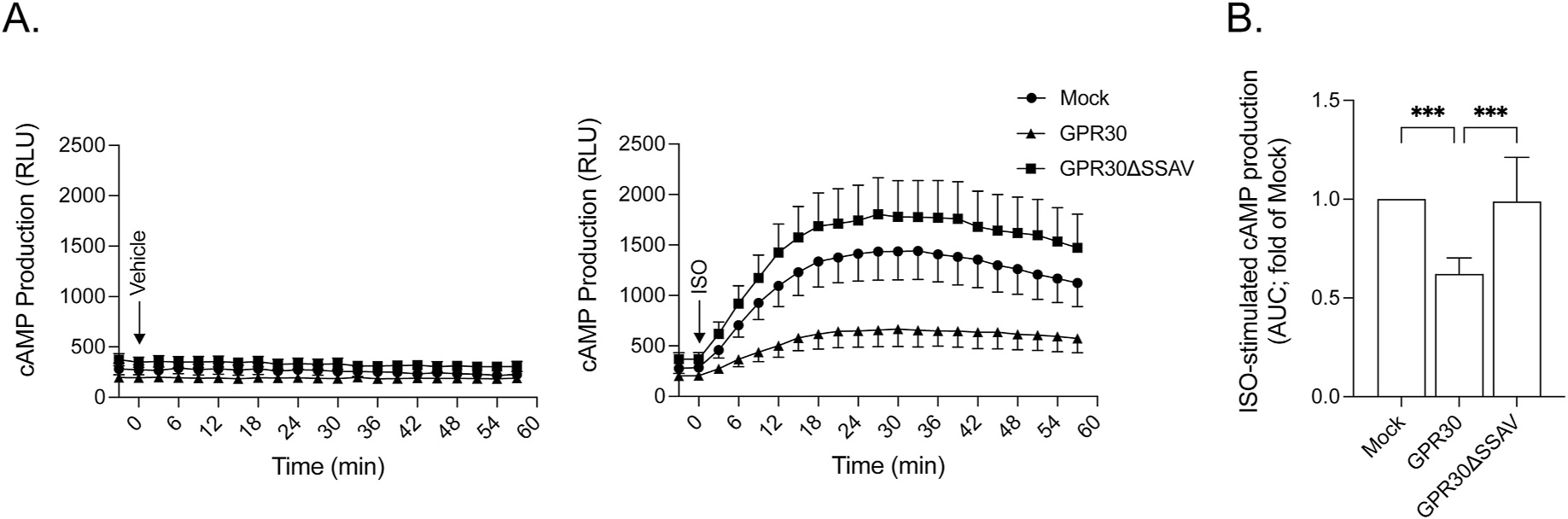
GPR30 PDZ-dependently inhibits β1AR-mediated cAMP production in MCF7 cells. In panel **A**, MCF7 cells transiently transfected with empty pcDNA3 plasmid (*Mock*) or plasmids containing GPR30 or GPR30ΔSSAV were treated without (*Vehicle*) or with 0.1 μM isoproterenol (*ISO*), and cAMP production was assayed in real time as luminescence with the GloSensor assay and presented as RLU with each data point being mean ± SEM of at least 3 experiment. In panel **B**, AUC was calculated for each treatment in panel **A**. The results are presented as fold of Mock with each data point being the mean ± SD of at least 3 experiments. ***, p < 0.001.

**Fig. 7.**
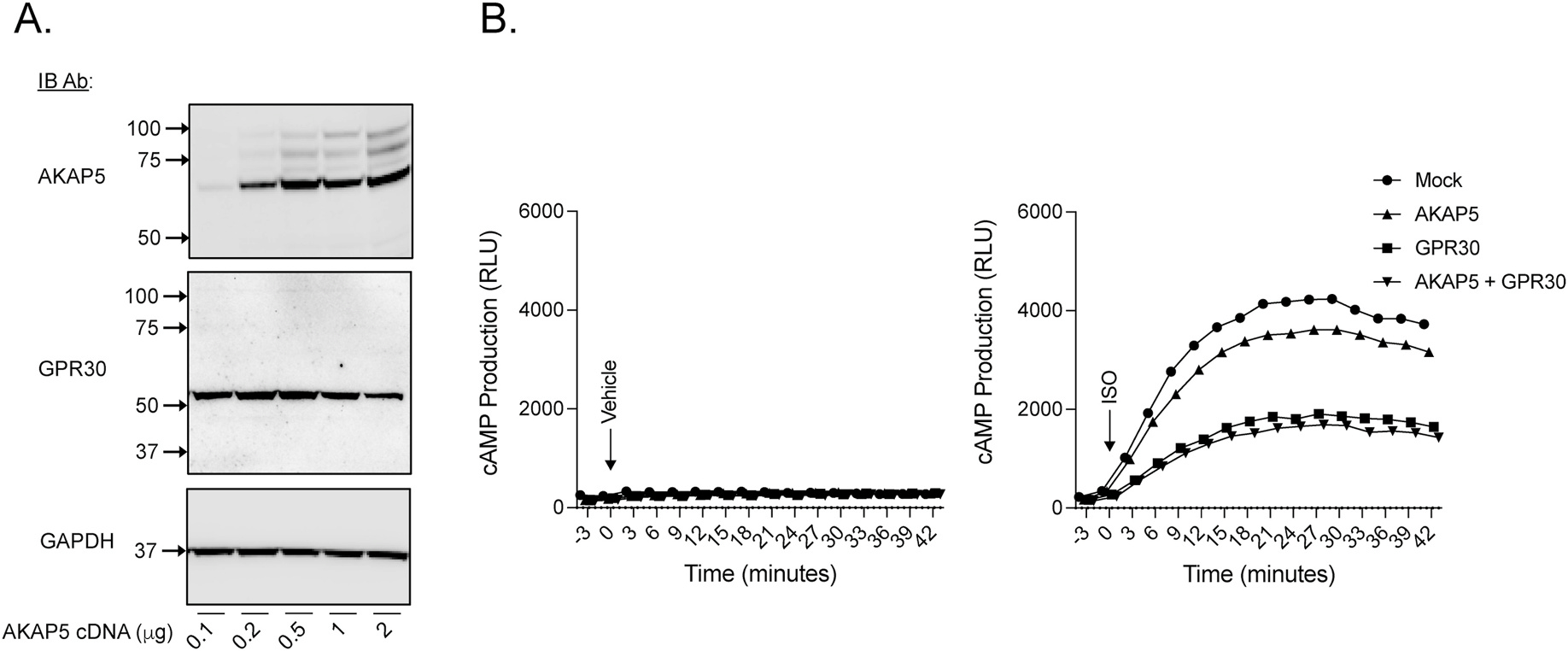
Involvement of AKAP5 in GPR30-dependent inhibition of β1AR-mediated cAMP production in MCF7 cells. In panel **A**, MCF7 cells transiently transfected with increasing amounts of plasmid containing AKAP5 (0.1–2 μg) were immunoblotted with AKAP5 Ab, stripped in reblotted with GPR30 Ab, and again stripped and reblotted with GAPDH Ab. In panel **B**, MCF7 cells transiently transfected with empty pcDNA3 plasmid (*Mock*) or plasmids containing AKAP5 or GPR30 or a combination of the two were treated without (*Vehicle*) or with 0.1 μM isoproterenol (*ISO*), and cAMP production was assayed in real time as luminescence with the GloSensor assay and presented as RLU with each data point being the mean of at least 3 experiments.

Fig. 6A and B shows that GPR30 expression constitutively inhibits endogenous β1AR signaling in a manner dependent on the C-terminal PDZ motif when expressed in MCF7 cells, consistent with our previous results in HEK293 cells [8]. To investigate if AKAP5 participates in this inhibition, AKAP5 was transiently expressed in MCF7 cells (Fig. 7A). AKAP5 expression by itself had a small but clear inhibitory effect on isoproterenol-stimulated β1AR signaling, and the effect did not appear to be additive with the inhibitory effect caused by GPR30 (Fig. 7B). Thus, GPR30 and AKAP5 seem to act at least in part through a common mechanism on β1AR signaling.

## 4. Discussion

Here, we show that GPR30 resides in a plasma membrane complex with β1AR, MAGUKs, and AKAP5 in MCF7 cells. Also, we provide evidence that this complex has a functional role in promoting constitutive PDZ-dependent GPR30 inhibition of β1AR-mediated cAMP production. We propose that this is a mechanism by which GPR30 constitutively regulates adrenergic signaling in breast cancer and possibly other pathophysiological systems.

GPR30 is a constitutively active G_i_-coupled receptor [8,10,11], though a very recent study showed that the receptor also can be ligand-activated [34]. The receptor has a complex subcellular distribution, with receptors identified in both various intracellular membranes and the plasma membrane. Considering the importance of plasma membrane-localized GPR30 for breast cancer outcome [15,16], we have made considerable efforts to determine the mechanism by which the receptor anchors in the plasma membrane and how this mechanism relates to constitutive receptor activity. Recent studies show that the anchoring of GPR30 in the plasma membrane is favored by an interaction between the receptor C-terminal type I PDZ motif with a plasma membrane-associated MAGUK scaffold protein [8,17–19] and in turn AKAP5 [8]. These plasma membrane interactions are also required for GPR30 to constitutively stimulate basal ERK1/2 activity, inhibit basal NFAT and Rac1 activity [10,11], and inhibit β1AR-mediated cAMP production [8]. Two MAGUKs have been reported to interact with GPR30, including PSD-95 [8,17,18] and SAP97 [8,19]. GPR30 also interacts with additional plasma membrane-localized effector proteins both PDZ-dependently, including Na^+^/H^+^ exchanger regulatory factor [35], and PDZ-independently, including RAMP3 [30]. Thus, GPR30 clearly has important functions in the plasma membrane.

β1AR is a G_s_-coupled receptor that resides in the plasma membrane to mediate adrenergic stimulation of cAMP production. The function of this receptor is best described in cardiomyocytes, where it also interacts via a C-terminal type I PDZ motif with a MAGUK and AKAP5, interactions important for receptor plasma membrane anchoring and critical for normal β1AR signaling [28,29]. GPR30 inhibits β1AR-mediated cAMP production in a PDZ-dependent manner [8]. A recent report showed that GPR30 also inhibits β1AR-mediated myocardial contractions and Ca^2+^ signaling [36].

Using immunoprecipitation, confocal immunofluorescence microscopy, and BRET with HEK293 cell expressing GPR30 and β1AR, we provide results arguing that these two receptors exist in close proximity in the plasma membrane. Several class A GPCR have been reported to physically interact by hetero-oligomerization [37]. Whether or not GPR30 and β1AR hetero-oligomerize is unclear as there are limitations with each of the methods used to assay such an interaction. Co-immunoprecipitation shows that the receptors are in close proximity, but immunoprecipitation involves detergent solubilization, which could lead to closely localized receptors being artificially forced together [38]. Furthermore, co-localization, as determined by confocal immunofluorescence imaging, reveals that two proteins have a similar subcellular localization, possibly in the same compartment, but not that they physically interact. BRET occurs when two proteins are within 10 nm, and this method has been used extensively to conclude that some class A GPCR physically interact [37]. However, this conclusion has been challenged as investigators using more advanced methods such as single molecule fluorescence resonance energy transfer (FRET) report that, while such receptors may be in close proximity, they do not actually physically interact [39]. Even though saturation BRET has been used extensively to study protein-protein interactions (37), technical issues with this approach has also been raised [40,41]. Nevertheless, our results from using multiple orthogonal methods and rigorous positive and negative controls strongly argue that GPR30 and β1AR reside in close proximity in a plasma membrane complex.

To address if GPR30, β1AR, MAGUKs, and AKAP5 also form a complex when expressed natively, we first identified that MCF7 breast cancer cells express all the proteins of interest. Using co-immunoprecipitation, we found that these proteins appear to form a complex also in these cells. Furthermore, GPR30 expression constitutively inhibits β1AR-mediated cAMP production in a PDZ-dependent manner in these cells. Finally, AKAP5 expression inhibits β1AR signaling in a non-additive manner with GPR30. Together, these results show that GPR30 and β1AR form a PDZ-independent complex through which GPR30 PDZ-dependently regulate β1AR signaling in breast cancer cells.

MAGUKs are scaffold proteins that anchor proteins in the plasma membrane through PDZ-dependent interactions. We initially hypothesized that the inhibitory GPR30 effect on β1AR signaling is a consequence of a PDZ-dependent complex of GPR30 and β1AR formed through their interactions with a common MAGUK. Using co-immunoprecipitation and BRET, we found that GPR30 and GPR30ΔSSAV, in which receptor PDZ motif had been removed, interacted to a more or less equal extent with β1AR. Thus, the receptor complex is not in itself PDZ-dependent and most likely not dependent on a common MAGUK.

Even though the exact mechanism for the PDZ-dependent GPR30 inhibition of β1AR signaling is presently unclear, it may be similar to that for GPR30 inhibition of FSHR-mediated cAMP production, which was recently reported to depend on receptor hetero-oligomerization and AKAP5 [13]. GPR30 has also been reported to hetero-oligomerize with KissR, yielding a decrease in Kiss1R at the cell surface [14]. Furthermore, GPR30 was shown to co-immunoprecipitate with the corticotropin releasing hormone receptor and 5HT_1a_ serotonin receptor, but no functional consequence was reported [17]. Thus, GPR30 appears to form plasma membrane complexes to constitutively regulate some other GPCR.

Several immunohistochemical studies have addressed the relationship between GPR30 and breast cancer outcome, but the results are inconsistent with the receptor conveying either better [42,43] or worse prognosis [15,16,44], or lacking any prognostic value [45] for breast cancer outcome. The complex receptor subcellular distribution appears to be one reason for this inconsistency. Indeed, we recently reported that receptor localization has direct pathophysiological consequences, with specifically plasma membrane-localized GPR30 predicting worse disease outcome [15,16]. The breast cancer cell background may be another factor that influences the GPR30 response. In support of this, we found that AKAP5 expression is substantially higher in MCF7 cells, in which GPR30 was reported to be apoptotic [42,46], than in SkBr3 cells, in which the receptor was reported to be proliferative [46]. Again, this is similar to the effect of GPR30 on FSHR, where the FSHR response is proliferative in the absence of GPR30, whereas it is apoptotic in the presence of GPR30, and the effect is dependent on AKAP5 expression [13]. It is therefore tempting to propose that the repertoire of GPR30 interacting proteins significantly effects the functions of this receptor in the cell.

The sympathetic nervous system influences breast cancer tumor microenvironment but the effect is complex and much knowledge is still lacking [22]. Preclinical studies in animal models suggest that sympathetic stimulation promotes tumor progression primarily through a β2AR subtype. A few studies have investigated the effect of βAR antagonists on breast cancer incidence and progression in humans. Retrospective studies suggest that antagonists can improve prognosis for breast cancer patients, and some results argue that the improvement is more pronounced in patients with triple-negative breast cancer [47,48]. However, most studies have used βAR subtype non-selective antagonists, which has made it difficult to conclude which adrenergic receptor subtype is involved in humans. Few, if any studies have been done regarding the role or prognostic value specifically of β1AR in breast cancer. However, noteworthy is that β1AR-selective antagonists used to treat hypertension dose-dependently increase the risk of developing breast cancer in hypertensive patients [23].

Based on the limited information available on the role of βAR in breast cancer, particularly β1AR, it is premature to draw any conclusions of the pathophysiological significance GPR30-promoted inhibition of β1AR-stimulated cAMP production in this disorder. Nevertheless, our results provide further insight into the mechanism by which GPR30 constitutively regulates some other GPCR and the importance of plasma membrane localization for these effects to occur. Specifically, we propose that GPR30 and β1AR form a PDZ-independent complex in native MCF7 cells through which GPR30 PDZ-dependently inhibits β1AR signaling via receptor interaction with a MAGUK and AKAP5. This complex may participate in regulating sympathetic effects on breast cancer progression.

## Supplementary Material

Supplemental Data

## Abbreviations:

GPR30: G protein-coupled receptor 30
GPER: G protein-coupled estrogen receptor
β1AR: β_1_-adrenergic receptor
β2AR: β_2_-adrenergic receptor
FSHR: follicle-stimulating hormone receptor
Kiss1R: kisspeptin receptor
GPCR: G protein-coupled receptor
ER: estrogen receptor α
E2: 17β-estradiol
MAGUK: membrane-associated guanylate kinase
PSD-95: postsynaptic density-95
SAP97: synapse-associated protein 97
AKAP5: protein kinase A anchoring protein 5
RAMP3: receptor activity modifying protein 3
PDZ: PSD-95/Discs-large/ZO-1 homology
GAPDH: glyceraldehyde-3-phosphate dehydrogenase
Ab: antibody
GFP: green fluorescent protein
eYFP: enhanced yellow fluorescent protein
rLuc: Renilla luciferase
BRET: bioluminescence resonance energy transfer
HEK: human embryonic kidney
DMEM: Dulbecco’s Modified Eagle’s Medium
FBS: fetal bovine serum
PBS: phosphate-buffered saline
WT: wild-type
SD: standard deviation
SEM: standard error of the mean
CI: confidence interval
AUC: area under curve
RLU: relative light units
