# Supplementary material for: G protein-coupled estrogen receptor (GPER)/GPR30 forms a complex with the β1-adrenergic receptor, a membrane-associated guanylate kinase (MAGUK) scaffold protein, and protein kinase A anchoring protein (AKAP) 5 in MCF7 breast cancer cells
Source: Arch Biochem Biophys. Author manuscript; Available in PMC 2024 Oct 16. (PMC11481754; doi:10.1016/j.abb.2024.109882)
Supplement: Supplemental Data [file NIHMS2025222-supplement-Supplemental_Data.pptx]

## Slide 1
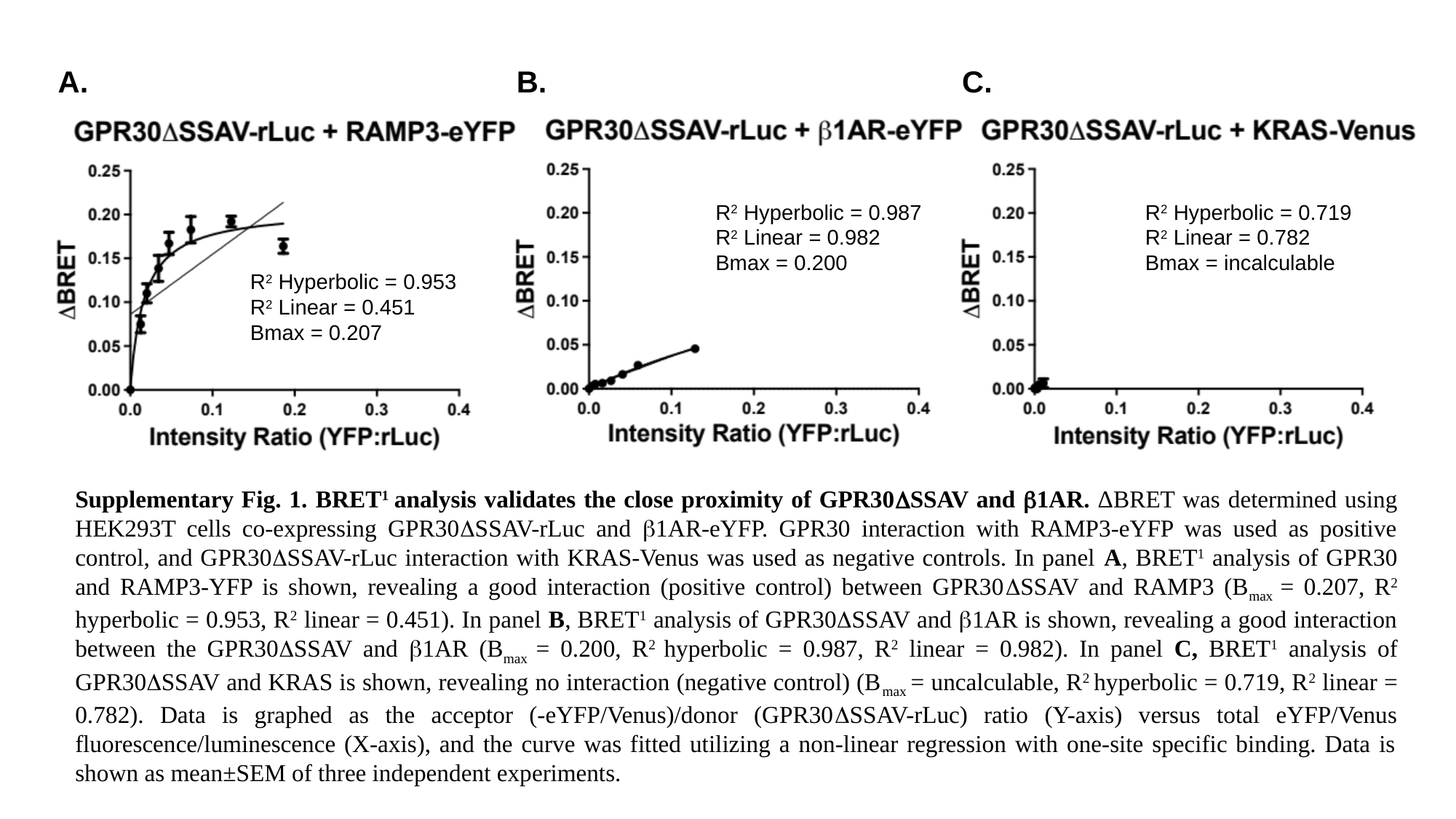

A.
R2 Hyperbolic = 0.953
R2 Linear = 0.451
Bmax = 0.207
B.
R2 Hyperbolic = 0.987
R2 Linear = 0.982
Bmax = 0.200
C.
R2 Hyperbolic = 0.719
R2 Linear = 0.782
Bmax = incalculable
Supplementary Fig. 1. BRET1 analysis validates the close proximity of GPR30DSSAV and β1AR. ΔBRET was determined using HEK293T cells co-expressing GPR30DSSAV-rLuc and β1AR-eYFP. GPR30 interaction with RAMP3-eYFP was used as positive control, and GPR30DSSAV-rLuc interaction with KRAS-Venus was used as negative controls. In panel A, BRET1 analysis of GPR30 and RAMP3-YFP is shown, revealing a good interaction (positive control) between GPR30DSSAV and RAMP3 (Bmax = 0.207, R2 hyperbolic = 0.953, R2 linear = 0.451). In panel B, BRET1 analysis of GPR30DSSAV and β1AR is shown, revealing a good interaction between the GPR30DSSAV and β1AR (Bmax = 0.200, R2 hyperbolic = 0.987, R2 linear = 0.982). In panel C, BRET1 analysis of GPR30DSSAV and KRAS is shown, revealing no interaction (negative control) (Bmax = uncalculable, R2 hyperbolic = 0.719, R2 linear = 0.782). Data is graphed as the acceptor (-eYFP/Venus)/donor (GPR30DSSAV-rLuc) ratio (Y-axis) versus total eYFP/Venus fluorescence/luminescence (X-axis), and the curve was fitted utilizing a non-linear regression with one-site specific binding. Data is shown as mean±SEM of three independent experiments.
